# Role of Caregivers in Remote Management of Patients With Type 2 Diabetes Mellitus: Systematic Review of Literature

**DOI:** 10.2196/46988

**Published:** 2023-09-11

**Authors:** Jun Jie Benjamin Seng, Meng Ferng Ryan Gwee, Mei Hui Amanda Yong, Yu Heng Kwan, Julian Thumboo, Lian Leng Low

**Affiliations:** 1 MOH Holding Private Limited Singapore Singapore; 2 SingHealth Regional Health System PULSES Centre Singapore Health Services Singapore Singapore; 3 Lee Kong Chian School of Medicine Singapore Singapore; 4 Department of Pharmacy Singapore General Hospital Singapore Singapore; 5 Department of Pharmacy National University of Singapore Singapore Singapore; 6 Program in Health Services and Systems Research Singapore Singapore; 7 Department of Rheumatology and Immunology Singapore General Hospital Singapore Singapore; 8 Department of Family Medicine and Continuing Care Singapore General Hospital Singapore Singapore; 9 Outram Community Hospital SingHealth Community Hospitals Singapore Singapore

**Keywords:** care, caregiver, diabetes, glucose monitoring, glucose, medication, mHealth, monitoring, patient care, quality of life, remote management, remote monitoring, systematic review, telehealth, telemedicine, type 2 diabetes mellitus, utilization

## Abstract

**Background:**

With the growing use of remote monitoring technologies in the management of patients with type 2 diabetes mellitus (T2DM), caregivers are becoming important resources that can be tapped into to improve patient care.

**Objective:**

This review aims to summarize the role of caregivers in the remote monitoring of patients with T2DM.

**Methods:**

We performed a systematic review in MEDLINE, Embase, Scopus, PsycINFO, and Web of Science up to 2022. Studies that evaluated the role of caregivers in remote management of adult patients with T2DM were included. Outcomes such as diabetes control, adherence to medication, quality of life, frequency of home glucose monitoring, and health care use were evaluated.

**Results:**

Of the 1198 identified citations, 11 articles were included. The majority of studies were conducted in North America (7/11, 64%) and South America (2/11, 18%). The main types of caregivers studied were family or friends (10/11, 91%), while the most common remote monitoring modalities evaluated were interactive voice response (5/11, 45%) and phone consultations (4/11, 36%). With regard to diabetes control, 3 of 6 studies showed improvement in diabetes-related laboratory parameters. A total of 2 studies showed improvements in patients’ medication adherence rates and frequency of home glucose monitoring. Studies that evaluated patients’ quality of life showed mixed evidence. In 1 study, increased hospitalization rates were noted in the intervention group.

**Conclusions:**

Caregivers may play a role in improving clinical outcomes among patients with T2DM under remote monitoring. Studies on mobile health technologies are lacking to understand their impact on Asian populations and long-term patient outcomes.

## Introduction

Remote management of patients refers to the use of digital technology for capturing a patient’s health-related data in real time, which is in turn transmitted and delivered to a health care professional to facilitate the management of a person’s medical condition [1]. The use of remote monitoring technologies for the delivery of patient care and health care services has gained popularity among policy administrators and health care professionals in the past few years [2]. These technologies offer a wide range of advantages, ranging from facilitating real-time assessment of patient outcome measures to reducing the number of hospital clinic visits, which reduces the burden on health care facilities [2]. In addition, they permit efficient delivery of patient care to patients residing in rural communities [3]. With the advent of the COVID-19 pandemic, the development and uptake of remote patient monitoring and telehealth technologies have been greatly accelerated [4]. Notably, the number of remote consultations has increased at least 50-fold compared to prepandemic times [5], while the market for telehealth has been estimated to exceed US $250 billion in the United States [6].

Type 2 diabetes mellitus (T2DM) is one of the leading metabolic diseases worldwide and afflicts nearly 500 million patients. It is associated with significant morbidity and mortality [7,8]. Health care expenditures from T2DM care and its associated complications amounted to over US $700 billion in 2017 and are expected to continue rising [7]. Given that achieving good diabetes control and regular self-monitoring of T2DM are paramount to slowing T2DM progression and reducing one’s risk of developing T2DM-related complications, it is unsurprising that research and development in diabetes-related remote monitoring technologies has been ramping up [9]. Various wearable and mobile technologies have been developed to target the wide range of laboratory and physical parameters, such as glucose levels, physical activity levels, and body weight, that are routinely assessed in diabetes care. For example, continuous wearable glucose monitors and insulin pumps have been developed to facilitate ease of monitoring and administration of insulin among patients with diabetes [10]. Technological advances have also led to increased functionality of smartphones, where inbuilt sensors such as the accelerometer, heart rate monitor, and gyroscope allow for assessment of physical activity level and caloric expenditure estimation [11].

Overall, remote monitoring technologies have demonstrated promising results among patients with T2DM. A recent review by Kitsiou et al [12] found that the use of mobile health technologies (mHealth) improved hemoglobin A_1c_ (HbA_1c_) by 0.8% among patients with T2DM. Another systematic review that examined the use of web-based remote monitoring systems showed similar improvements in glycemic control in patients with T2DM [13]. Despite the potential benefits of remote patient monitoring, the real-world adoption of these related technologies has lagged due to patients’ perceived barriers [14]. In a study by Foong et al [15] that evaluated themes affecting uptake of digital technology for diabetes-related foot ulcers, common barriers identified included knowledge-related barriers such as lack of technological savviness and physical limitations, for example, difficulty with reading on a smartphone, lack of dexterity with manipulation of devices, and the need for an assistant to take photos [15]. The use of other remote monitoring technologies and devices is likely to encounter similar barriers and would require regular education and training [10]. Caregivers could aid in filling these gaps to support and facilitate adherence to the usage of such technologies, especially among older people who may be less technologically literate. A qualitative study that evaluated the role of family caregivers in diabetes care found that family support played an important role in supporting patients’ use of health information technologies and patient web portals [16]. Another study that evaluated a combined program involving telemonitoring and informal caregiver involvement among patients with T2DM showed a significant improvement in patients’ adherence to medication, foot examinations, and regular glucose monitoring [17].

To the best of our knowledge, no review has been undertaken to evaluate the role of caregivers in remote monitoring of patients with T2DM. The reviews available in the literature have only evaluated outcomes associated with remote patient monitoring [18,19], telehealth tools and interventions to support family caregivers in patient care [20], and caregivers’ experiences with remote monitoring technologies [21] in other patient populations, such as patients with dementia or hypertension. Within patients with T2DM, the existing literature is limited to reviews that examined barriers affecting uptake of remote monitoring technologies [22], outcomes associated with remote monitoring technologies [23], and caregivers and patients’ perspectives toward remote monitoring technologies [24]. As such, the objective of this study was to evaluate and summarize the role of caregivers in the remote management of patients with T2DM.

## Methods

### Protocol and Registration

The protocol for this review was registered on the Open Science Framework (JCWTD) and reported in accordance with the PRISMA (Preferred Reporting Items for Systematic Reviews and Meta-Analyses) checklist.

### Information Sources and Search Strategy

We conducted a systematic review in 5 major literature databases, which included MEDLINE, Embase, CINAHL, PsycINFO, and Web of Science. No restriction was imposed on the start date of the search, and the review was current as of February 28, 2022. The search strategy used key terminologies related to T2DM, caregivers or caregiving, and remote management of patients (Multimedia Appendix 1 [17,25-34]). The search terms used were adapted from systematic reviews that evaluated remote management or caregiving in other patient populations [23,35-37].

### Definitions

#### Caregivers

For this review, 4 main categories of caregivers, which included informal, volunteer, professional, and independent or private caregivers, were evaluated [38]. Informal caregivers are family members or friends who provide typically unpaid care to a patient with whom they have a personal relationship [39]. Professional caregivers typically work for an agency and provide home- or facility-based medical or nonmedical care, while independent or private caregivers are typically hired directly by the patient’s family to provide medical or nonmedical care [38].

#### Remote Monitoring Technologies

Remote monitoring technologies typically comprise of three key elements: (1) electronic transmission of health-related information, for example, blood pressure or self-reported measures, across 2 different geographical locations; (2) use of electronic devices in a patient’s home, mobile devices on patients (eg, computers and handphones); and (3) feedback from a health care professional who provides tailored advice to the patient or automated feedback delivered based on a predefined algorithm [40]. Examples of these technologies may include telephone consultations and video conferencing, as well as the use of digital devices and wearables, for example, smartwatches, and electronic networks for the delivery of health services or information [1,41].

### Eligibility Criteria and Selection Process

We included full-text articles in English that assessed the role of caregivers or caregiving in remote management of patients with T2DM. Using the “patient, intervention, comparator, and outcomes” (PICO) guide, the patient population of interest was patients with T2DM, while interventions included the provision or inclusion of caregivers or caregiving in the remote monitoring of patients with T2DM. Comparator groups for the included studies included patients with T2DM who received usual care or other remote monitoring interventions without caregivers. Studies that evaluated only the role of caregivers or remote management of patients with T2DM separately, as well as studies that included patients with type 1 diabetes mellitus or maturity-onset diabetes in the young, were excluded. We also excluded case reports, series, irrelevant systematic reviews, and meta-analyses.

All references and abstracts extracted from the databases were exported to the EndNote (version X9; Clarivate). Duplicate citations were removed, and the screening of the titles, abstracts, and full text of the retrieved citations was performed by 2 independent reviewers (JJBS and MFRG) to identify relevant articles for inclusion. All disagreements during the initial screening process were discussed, and any unresolved disagreements were arbitrated by a third independent reviewer (MHAY). Additionally, hand searching of references within the included studies was performed to identify other relevant studies.

### Data Collection Process and Data Items

Information from included citations was collated in a standardized Excel (Microsoft Corp) worksheet by 2 independent reviewers. We conducted an initial pilot data extraction for the first 15 citations to ensure the accuracy of data extraction. The study details extracted included the study title, publication year, sample size, characteristics of the patient population, details related to caregivers, and modality of remote monitoring. With regard to outcomes of interest, the Economic, Clinical and Humanistic Outcome model proposed by Kozma et al [42] was adapted. It is an integrated approach that considers economic, clinical, and humanistic outcomes in the allocation of health care resources. The outcomes from the included studies were broadly classified into 3 main categories: clinical, humanistic, and economic outcomes based on the model. Clinical outcomes were further subdivided into T2DM related, non-T2DM related, and other health outcomes. Humanistic outcomes encompassed measures related to the patient’s health-related quality of life, other patient-reported outcomes, and behavioral changes [42]. Economic outcomes included health care–related expenditures and health resource use.

### Studying the Risk of Bias Assessment and Reporting Bias Assessment

In this review, the study quality assessment tools from the National Heart, Lung, and Blood Institute were used to evaluate the risk of bias among the included studies [43]. A total of 2 independent reviewers (JJBS and MFRG) evaluated the methodological quality and risk of bias among the included studies. The checklists consist of items that assess each individual study’s risk of bias associated with the research question, patient population recruitment, outcome evaluation, and patient dropout percentages. All disagreements in the risk of bias assessment were arbitrated with a third independent reviewer (MHAY).

### Effect Measures and Synthesis Methods

Details related to the characteristics of all included studies are available in Multimedia Appendix 1 [17,25-34]. Descriptive statistics were used to summarize the characteristics of the included studies. Continuous variables were reported as the mean (SD), while categorical variables were reported as n (%). To improve the completeness of this review, the authors of studies with missing information were contacted. A total of 2 separate email reminders were sent out 2 weeks apart, and information that could not be retrieved was labeled as unavailable. Data imputation was not performed in this review.

To evaluate if meta-analyses could be performed for this review, 2 independent reviewers (JJBS and MFRG) examined the clinical and methodological heterogeneity of the included studies. Clinical heterogeneity looks at the differences in the characteristics of patient population and outcomes. On the other hand, methodological heterogeneity refers to variation in study design and risk of bias. Due to the expected heterogeneity of the included studies, meta-analyses were not performed. A narrative synthesis was provided for the role of caregivers in the remote management of patients with T2DM.

## Results

**Figure 1:**
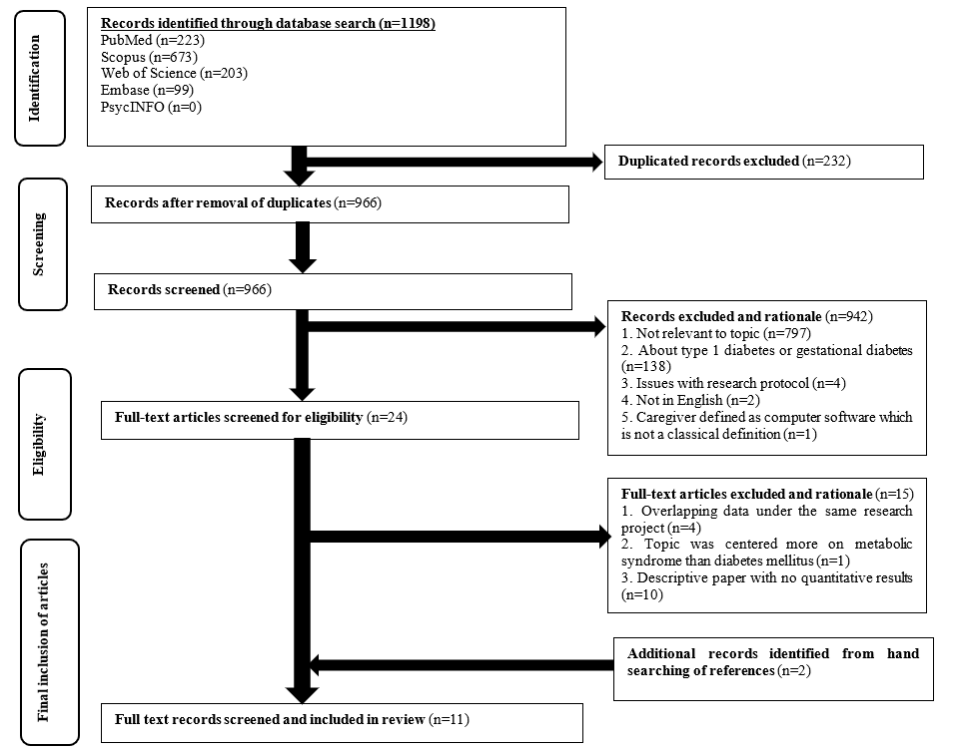
Flowchart for inclusion of articles.

A total of 11 articles were included in this review from the initial 1198 articles identified (Figure 1). The percentage of articles agreed upon between MFRG and JJBS during the screening of articles was 91%. All disagreements were resolved.

### Study Characteristics

Table 1 shows the characteristics of the studies in this review. The majority of the studies (6/11, 55%) were conducted between 2001 and 2010. Most of the studies were conducted in North America (7/11, 64%), and the most common study design was a randomized controlled trial (5/11, 46%). Most studies were conducted in a primary health care setting (8/11, 73%), and all studies used primary data sources (n=11). The majority of the caregivers in the studies involved family members or friends (10/11, 91%), while the most common remote monitoring modalities were interactive voice response (5/11, 46%) and phone consultations (4/11, 36%). With regard to the risk of bias, 3 studies were noted to have a moderate risk of bias, while the remaining studies were assessed to have a low risk of bias (Multimedia Appendix 1 [17,25-34]).

Table S1 in Multimedia Appendix 2 [17,25-34] shows a summary of study details related to the 11 studies included. The follow-up period across studies ranged from 3 months to 3 years.

**Table 1:** Characteristics of included studies (N=11).

Characteristics of studies		Frequency, n (%)
**Year of study**		
	Between 2001 and 2010	6 (55)
	Between 2011 and 2021	5 (46)
**Continent of study**		
	North America	7 (64)
	South America	2 (18)
	Asia	1 (9)
	Europe	1 (9)
**Country of study**		
	United States of America	6 (55)
	Bolivia	1 (9)
	Brazil	1 (9)
	China	1 (9)
	United Kingdom	1 (9)
	Multiple countries	1 (9)
**Study design**		
	Randomized controlled trial	5 (46)
	Observational study	4 (36)
	Retrospective cohort study	2 (18)
**Patient population, n**		
	1-100	3 (27)
	101-200	1 (9)
	201-300	2 (18)
	301-400	3 (27)
	>500	2 (18)
**Study setting (health care)**		
	Primary	8 (73)
	Tertiary	3 (27)
**Data source**		
	Primary	11 (100)
**Type of caregivers**		
	Family or friends	10 (91)
	Others	1 (9)
**Remote monitoring modality**		
	Interactive voice response	5 (46)
	Phone consultations	4 (36)
	Text messages	1 (9)
	Apps (web or mobile)	1 (9)

### Clinical Outcomes

#### Laboratory Parameters

Table S2 in Multimedia Appendix 3 [17,25-34] shows the types of outcomes and the results of the studies. A total of 6 studies explored the impact on diabetes-related parameters. Of these 6 studies, 3 showed significant improvements in diabetes-related parameters (*P≤*.05) for the intervention group. Patients in the intervention arm showed lower HbA_1c_ compared with the control group as well as improvements in parameters such as fasting blood glucose and self-reported glucose reading compared with the control group [17,25,26]. On the other hand, the other 3 studies showed no significant improvements in diabetes-related parameters (*P*>.05) for the intervention group in areas including HbA_1c_, fasting plasma glucose, and the lipid panel [28-30].

#### Medication Adherence

A total of 2 studies assessed self-reported adherence to diabetes medications. One of the studies reported a 19% improvement in medication adherence in the intervention group over the control group (adjusted odds ratio [AOR] 1.19; *P*=.03) while the other reported a significant improvement in the Morisky Medication Adherence Scale before and after intervention (95% CI −0.42 to −0.18; *P*<.001) [17,25].

### Humanistic Outcomes

#### Patients’ Diabetes-Related Symptoms and Distress

All 3 studies that explored diabetes-related symptoms and distress showed that the intervention group had significantly better diabetes-related symptoms and distress in terms of general health, days spent in bed due to illnesses, or diabetes-related distress (*P*≤.05) [17,25,31].

#### Patients’ Quality of Life

A total of 2 studies explored the impact on patients’ quality of life [17,29]. One of them showed significant improvements in patients’ quality of life in terms of physical function (*P*≤.05) in the intervention group, while the other study showed no significant improvements in quality of life (*P*>.05).

#### Patient Satisfaction

A study examined the impact of a patient's perceived satisfaction with the program through follow-up survey questions. The majority of the patients (89%) reported being “very satisfied” with the program that they were enrolled in (*P*=.04) [31].

#### Compliance to Remote Monitoring

A total of 5 studies explored the impact of remote monitoring on patients’ compliance. All 5 studies showed significant increases in the patients’ compliance to remote monitoring in areas such as call completion rates, diabetes course completion rates, and blood glucose and blood pressure monitoring [17,26,29,32,33].

### Economic Outcomes

A study explored the impact on patients’ health care use. The study showed significantly higher rates of 12-month hospitalizations compared with the control group (*P*≤.05) [34].

## Discussion

### Overview

To the best of our knowledge, this is the first systematic review that has evaluated the role of caregivers in the remote management of T2DM. Current evidence in the literature appears to suggest a positive impact of caregivers on T2DM with regards to clinical, economic, and humanistic outcomes ranging from diabetes-related parameters to patients’ compliance to monitoring.

The positive impact of caregivers on remote monitoring of T2DM is likely multifactorial. Studies have shown that common barriers to remote monitoring include patient-related factors such as health literacy, technology-related barriers, and challenging patient experiences with remote monitoring technologies [44]. Through the partnership with caregivers, knowledge gaps related to technical aspects of remote monitoring technologies and T2DM can be bridged, which in turn aids in improving patients’ adherence to medication and remote monitoring services [45]. This is important in the context of varied levels of electronic health literacy among older patients, which often impair their ability to comply with remote monitoring technologies [46]. In addition, patients with poor language skills, poor digital literacy skills, and equipment deficiencies are often excluded from remote monitoring programs and studies due to technological and logistical limitations [47]. With caregivers serving as potential avenues to bridge some of the modifiable gaps, there is a potential role to allow for the expansion of remote monitoring technologies to these previously excluded patient populations if suitable caregivers can be identified to empower patients.

With regard to the mixed evidence related to improvements in T2DM-related laboratory parameters, there are several reasons that may account for the lack of statistical differences in T2DM parameters in half of the studies. For example, the study by Burner et al [29] was limited by the study sample size as it was intended to be a feasibility study and was not adequately powered to evaluate differences in glycemic control between the intervention and control groups. Likewise, the study by Gambling and Long [30] sought to qualitatively evaluate telephone-based support for patients with T2DM and recruited only 9 patients. Another postulated reason could be due to underestimation of the effects of an intervention when it is offered alongside routine care, which has been seen in other studies evaluating the education of patients with T2DM [48]. Hence, larger studies are required to confirm and evaluate the impact caregivers have on the clinical outcomes of patients with T2DM.

Interestingly, the study by Wakefield Vaughan-Sarrazin [34] showed an increased number of hospitalizations among patients with T2DM on remote monitoring with caregivers compared with the control group. In this study, one of the key findings was a significantly higher caregiver burden and strain in the intervention group. This highlights the importance of recognizing the potential impacts on caregivers when engaging them in the remote monitoring of patients with T2DM. A review performed by Doherty et al [49] found that caregivers also have their own unique set of needs, which center around psychosocial support to maintain normalcy, maintaining their social lives, and support with navigating health care systems. Likewise, caregivers may also suffer from underlying medical conditions, and studies have shown that poor social support and depressive symptoms are associated with poor outcomes in caregiving [50]. Familial strife may also ensue from the overload of caregiving tasks, limited financial resources, and underappreciation of the work rendered by caregivers [51]. With the looming shortage of caregivers and decreasing caregiver-to-patient ratio globally due to declining fertility rates [52], developing strategies to ensure efficient and safe enlistment of caregivers in remote monitoring of patients with T2DM will be crucial. Some interventions suggested before the initiation of remote monitoring strategies include having caregivers attend clinics to allow physicians to formally assess them for their suitability for remote monitoring programs [53].

In this review, only 2 studies evaluated the potential of mHealth or web-based apps with caregivers. Nudge strategies, which encompass reminders, gamification, and social modeling approaches to drive positive health-related behaviors, have been increasingly recognized as a potential solution to improving diabetes control [54]. With advances in information and communication-related technology, there is a growing number of multifunctional social media and mobile apps that can deliver scalable behavior nudges for patients with T2DM [55]. For example, mHealth applications on a widely used Chinese social media platform, WeChat, allow for the sharing of blood pressure readings with patients’ caregivers, which allows them to aid in nudging patients [56]. Future studies should look into exploring the role of mHealth apps and the involvement of caregivers in diabetes care.

### Limitations of This Review

The results of this systematic review should be interpreted in the context of the following limitations: due to significant heterogeneity in the training of caregivers and outcomes assessed across the studies, meta-analyses could not be performed. Future reviews should consider performing meta-analyses of the developing literature on remote monitoring technologies used in patients with T2DM and the role of caregivers. Another limitation was the lack of standardized outcomes assessed across studies. Future studies should look into developing a core set of outcomes for studies examining remote monitoring technologies for diabetes patients. Currently, generic instruments such as the CONSORT-eHealth checklist have been developed, but disease-specific instruments are required for the assessment of patients with T2DM [57]. This will in turn aid in facilitating meaningful cross-comparisons of different caregivers and their impact on remote monitoring of patients with T2DM. Lastly, the follow-up period of the studies included in this review is generally short. One of the main challenges with the use of remote monitoring technologies is their increasing attrition rates with time, which can be as high as 50% [58,59]. More longitudinal studies are required to assess the role of caregivers in reducing patient attrition rates and their long-term impact on patients with T2DM who are remotely monitored.

### Conclusions

This review has shown that caregivers may play a role in improving clinical outcomes among patients with T2DM under remote monitoring. More studies are required to understand the impact of caregivers on less studied populations, such as Asian populations, and long-term patient outcomes.

## Appendix

Multimedia Appendix 1 Details of full search strategy and risk of bias assessment for included studies.

Multimedia Appendix 2 Summary of the 11 studies included into the systematic review.

Multimedia Appendix 3 Summary of the outcome measures reported from the 11 studies included into the review.

## Abbreviations

AOR: adjusted odds ratio
HbA1c: hemoglobin A1c
mHealth: mobile health
PICO: patient, intervention, comparator, and outcomes
PRISMA: Preferred Reporting Items for Systematic Reviews and Meta-Analyses
T2DM: type 2 diabetes mellitus

## Data Availability

All data generated or analyzed during this study are included in this published article and its Multimedia Appendices.
