# Supplementary material for: Role of Caregivers in Remote Management of Patients With Type 2 Diabetes Mellitus: Systematic Review of Literature
Source: J Med Internet Res. 2023 Sep 11;25:e46988. doi: 10.2196/46988 (PMC10520771; doi:10.2196/46988)
Supplement: Multimedia Appendix 1 [file jmir_v25i1e46988_app1.docx]

Supplementary File 1. Details of full search strategy

**Search Terms**

**1) Medline®**

Search strategy: #1 AND #2 AND #3

| **S/No.** | **Key themes** | **Terms** |
| --- | --- | --- |
| 1 | Type 2 Diabetes Mellitus | ("diabetes mellitus"[MeSH Terms] OR ("diabetes"[All Fields] AND "mellitus"[All Fields]) OR "diabetes mellitus"[All Fields] OR ("diabetes mellitus, type 2"[MeSH Terms] OR "type 2 diabetes mellitus"[All Fields] OR "diabetes mellitus type 2"[All Fields]) OR "T2DM"[All Fields] OR ("diabetes mellitus, type 2"[MeSH Terms] OR "type 2 diabetes mellitus"[All Fields]) OR ("diabetes mellitus, type 2"[MeSH Terms] OR "type 2 diabetes mellitus"[All Fields] OR "niddm"[All Fields] OR "niddms"[All Fields]) OR ("diabetes mellitus, type 2"[MeSH Terms] OR "type 2 diabetes mellitus"[All Fields] OR ("non"[All Fields] AND "insulin"[All Fields] AND "dependent"[All Fields] AND "diabetes"[All Fields] AND "mellitus"[All Fields]) OR "non insulin dependent diabetes mellitus"[All Fields])) |
| 2 | Caregivers | ("caregiver s"[All Fields] OR "caregivers"[MeSH Terms] OR "caregivers"[All Fields] OR "caregiver"[All Fields] OR "caregiving"[All Fields] OR "caregiver*"[All Fields] OR ("caregivers"[MeSH Terms] OR "caregivers"[All Fields] OR "carer"[All Fields] OR "carers"[All Fields] OR "carer s"[All Fields]) OR ("familialities"[All Fields] OR "familiality"[All Fields] OR "familially"[All Fields] OR "familials"[All Fields] OR "familie"[All Fields] OR "family"[MeSH Terms] OR "family"[All Fields] OR "familial"[All Fields] OR "families"[All Fields] OR "family s"[All Fields] OR "familys"[All Fields]) OR ("caregivers"[MeSH Terms] OR "caregivers"[All Fields] OR ("care"[All Fields] AND "giver"[All Fields]) OR "care giver"[All Fields]) OR ("caregivers"[MeSH Terms] OR "caregivers"[All Fields] OR ("spouse"[All Fields] AND "caregiver"[All Fields]) OR "spouse caregiver"[All Fields]) OR ("caregivers"[MeSH Terms] OR "caregivers"[All Fields] OR ("family"[All Fields] AND "caregiver"[All Fields]) OR "family caregiver"[All Fields])) |
| 3 | Remote management | ("telemedicine"[MeSH Terms] OR "telemedicine"[All Fields] OR "telemedicine s"[All Fields] OR ("telehealth s"[All Fields] OR "telemedicine"[MeSH Terms] OR "telemedicine"[All Fields] OR "telehealth"[All Fields]) OR ("telemonitor"[All Fields] OR "telemonitored"[All Fields] OR "telemonitoring"[All Fields] OR "telemonitors"[All Fields]) OR "tele-monitor"[All Fields] OR ("telemonitor"[All Fields] OR "telemonitored"[All Fields] OR "telemonitoring"[All Fields] OR "telemonitors"[All Fields]) OR "Tele-monitoring"[All Fields] OR ("telemedicine"[MeSH Terms] OR "telemedicine"[All Fields] OR "ehealth"[All Fields]) OR (("electronical"[All Fields] OR "electronically"[All Fields] OR "electronics"[MeSH Terms] OR "electronics"[All Fields] OR "electronic"[All Fields]) AND ("health"[MeSH Terms] OR "health"[All Fields] OR "health s"[All Fields] OR "healthful"[All Fields] OR "healthfulness"[All Fields] OR "healths"[All Fields])) OR "e-health"[All Fields] OR ("telemedicine"[MeSH Terms] OR "telemedicine"[All Fields] OR ("mobile"[All Fields] AND "health"[All Fields]) OR "mobile health"[All Fields]) OR (("remote"[All Fields] OR "remotely"[All Fields] OR "remoteness"[All Fields] OR "remotes"[All Fields]) AND ("manage"[All Fields] OR "managed"[All Fields] OR "management s"[All Fields] OR "managements"[All Fields] OR "manager"[All Fields] OR "manager s"[All Fields] OR "managers"[All Fields] OR "manages"[All Fields] OR "managing"[All Fields] OR "managment"[All Fields] OR "organization and administration"[MeSH Terms] OR ("organization"[All Fields] AND "administration"[All Fields]) OR "organization and administration"[All Fields] OR "management"[All Fields] OR "disease management"[MeSH Terms] OR ("disease"[All Fields] AND "management"[All Fields]) OR "disease management"[All Fields])) OR (("remote"[All Fields] OR "remotely"[All Fields] OR "remoteness"[All Fields] OR "remotes"[All Fields]) AND ("monitor s"[All Fields] OR "monitorable"[All Fields] OR "monitored"[All Fields] OR "monitoring"[All Fields] OR "monitoring s"[All Fields] OR "monitoring, physiologic"[MeSH Terms] OR ("monitoring"[All Fields] AND "physiologic"[All Fields]) OR "physiologic monitoring"[All Fields] OR "monitor"[All Fields] OR "monitorings"[All Fields] OR "monitorization"[All Fields] OR "monitorize"[All Fields] OR "monitorized"[All Fields] OR "monitors"[All Fields])) OR ("remote consultation"[MeSH Terms] OR ("remote"[All Fields] AND "consultation"[All Fields]) OR "remote consultation"[All Fields]) OR ("mhealth s"[All Fields] OR "telemedicine"[MeSH Terms] OR "telemedicine"[All Fields] OR "mhealth"[All Fields]) OR "m-health"[All Fields] OR ("lancet digit health"[Journal] OR "digit health"[Journal] OR ("digital"[All Fields] AND "health"[All Fields]) OR "digital health"[All Fields]) OR ("telematic"[All Fields] OR "telematics"[All Fields]) OR "telemanagement"[All Fields] OR "Tele-management"[All Fields] OR "tele-nursing"[All Fields] OR ("telenursing"[MeSH Terms] OR "telenursing"[All Fields]) OR "teleservice"[All Fields] OR "tele-service"[All Fields] OR "tele-care"[All Fields] OR "telecare"[All Fields] OR "Tele-home"[All Fields] OR "telehome"[All Fields] OR "tele conferenc*"[All Fields] OR "teleconferenc*"[All Fields] OR ("remote consultation"[MeSH Terms] OR ("remote"[All Fields] AND "consultation"[All Fields]) OR "remote consultation"[All Fields] OR "teleconsultation"[All Fields] OR "teleconsultations"[All Fields] OR "teleconsult"[All Fields] OR "teleconsultant"[All Fields] OR "teleconsultants"[All Fields] OR "teleconsultative"[All Fields] OR "teleconsulting"[All Fields] OR "teleconsults"[All Fields]) OR "Tele-consult"[All Fields] OR ("telecommunications"[MeSH Terms] OR "telecommunications"[All Fields] OR "telecommunication"[All Fields]) OR "tele-communication"[All Fields] OR (("remote sens basel"[Journal] OR "int j remote sens"[Journal] OR ("remote"[All Fields] AND "sensing"[All Fields]) OR "remote sensing"[All Fields]) AND "technolog*"[All Fields]) OR "wearable*"[All Fields] OR ("wearable electronic devices"[MeSH Terms] OR ("wearable"[All Fields] AND "electronic"[All Fields] AND "devices"[All Fields]) OR "wearable electronic devices"[All Fields] OR ("wearable"[All Fields] AND "device"[All Fields]) OR "wearable device"[All Fields]) OR (("wearability"[All Fields] OR "wearable"[All Fields] OR "wearables"[All Fields]) AND ("sensor"[All Fields] OR "sensor s"[All Fields] OR "sensoric"[All Fields] OR "sensorics"[All Fields] OR "sensoring"[All Fields] OR "sensorization"[All Fields] OR "sensorized"[All Fields] OR "sensors"[All Fields]))) |

**2) Embase®**

Search strategy: #1 AND #2 AND #3

| **S/No.** | **Key themes** | **Terms** |
| --- | --- | --- |
| 1 | Type 2 Diabetes Mellitus | ('diabetes mellitus':ti,ab,kw OR 'diabetes mellitus, type 2':ti,ab,kw OR t2dm:ti,ab,kw OR 'type 2 diabetes mellitus':ti,ab,kw OR niddm:ti,ab,kw OR 'non-insulin dependent diabetes mellitus':ti,ab,kw) |
| 2 | Caregivers | (caregiver:ti,ab,kw OR caregiver*:ti,ab,kw OR carer:ti,ab,kw OR family:ti,ab,kw OR 'care giver':ti,ab,kw OR 'spouse caregiver':ti,ab,kw OR 'family caregiver':ti,ab,kw) |
| 3 | Remote management | (telemedicine:ti,ab,kw OR telehealth:ti,ab,kw OR telemonitor:ti,ab,kw OR 'tele monitor':ti,ab,kw OR telemonitoring:ti,ab,kw OR 'tele monitoring':ti,ab,kw OR ehealth:ti,ab,kw OR 'electronic health':ti,ab,kw OR 'e health':ti,ab,kw OR 'mobile health':ti,ab,kw OR 'remote management':ti,ab,kw OR 'remote monitoring':ti,ab,kw OR 'remote consultation':ti,ab,kw OR mhealth:ti,ab,kw OR 'm health':ti,ab,kw OR 'digital health':ti,ab,kw OR telematic:ti,ab,kw OR 'tele matic':ti,ab,kw OR telemanagement:ti,ab,kw OR 'tele management':ti,ab,kw OR 'tele nursing':ti,ab,kw OR telenursing:ti,ab,kw OR teleservice:ti,ab,kw OR 'tele service':ti,ab,kw OR 'tele care':ti,ab,kw OR telecare:ti,ab,kw OR 'tele home':ti,ab,kw OR telehome:ti,ab,kw OR 'tele conferenc*':ti,ab,kw OR teleconferenc*:ti,ab,kw OR teleconsult:ti,ab,kw OR 'tele consult':ti,ab,kw OR telecommunication:ti,ab,kw OR 'tele communication':ti,ab,kw OR 'remote sensing technolog*':ti,ab,kw OR wearable*:ti,ab,kw OR 'wearable device':ti,ab,kw OR 'wearable sensor':ti,ab,kw) |

**3) SCOPUS®**

Search strategy: #1 AND #2 AND #3

| **S/No.** | **Key themes** | **Terms** |
| --- | --- | --- |
| 1 | Type 2 Diabetes Mellitus | TITLE-ABS-KEY ( ( ( diabetes AND mellitus ) OR ( diabetes AND mellitus, AND type 2 ) OR ( t2dm ) OR ( type 2 diabetes AND mellitus ) OR ( niddm ) OR ( non-insulin AND dependent AND diabetes AND mellitus ) ) |
| 2 | Caregivers | TITLE-ABS-KEY ( ( caregiver ) OR ( caregiver* ) OR ( carer ) OR ( family ) OR ( care AND giver ) OR ( spouse AND caregiver ) OR ( family AND caregiver ) ) |
| 3 | Remote management | TITLE-ABS-KEY ( ( ( telemedicine ) OR ( telehealth ) OR ( telemonitor ) OR ( tele-monitor ) OR ( telemonitoring ) OR ( tele-monitoring ) OR ( ehealth ) OR ( electronic AND health ) OR ( e-health ) OR ( mobile AND health ) OR ( remote AND management ) OR ( remote AND monitoring ) OR ( remote AND consultation ) OR ( mhealth ) OR ( m-health ) OR ( digital AND health ) OR ( telematic ) OR ( tele-matic ) OR ( telemanagement ) OR ( tele-management ) OR ( tele-nursing ) OR ( telenursing ) OR ( teleservice ) OR ( tele-service ) OR ( tele-care ) OR ( telecare ) OR ( tele-home ) OR ( telehome ) OR ( tele-conferenc* ) OR ( teleconferenc* ) OR ( teleconsult ) OR ( tele-consult ) OR ( telecommunication ) OR ( tele-communication ) OR ( remote AND sensing AND technolog* ) OR ( wearable* ) OR ( wearable AND device ) OR ( wearable AND sensor ) ) ) ) |

**4) PsycInfo®**

| **S/No.** | **Key themes** | **Terms** |
| --- | --- | --- |
| 1 | Type 2 Diabetes Mellitus | ((Diabetes mellitus) OR (Diabetes mellitus, type 2) OR (T2DM) OR (Type 2 diabetes mellitus) OR (NIDDM) OR (non-insulin dependent diabetes mellitus)) |
| 2 | Caregivers | ((Caregiver) OR (caregiver*) OR (carer) OR (family) OR (care giver) OR (spouse caregiver) OR (family caregiver)) |
| 3 | Remote management | ((Telemedicine) OR (telehealth) OR (Telemonitor) OR (tele-monitor) OR (Telemonitoring) OR (Tele-monitoring) OR (ehealth) OR (electronic health) OR (e-health) OR (mobile health) OR (remote management) OR (remote monitoring) OR (remote consultation) OR (mhealth) or (m-health) OR (digital health) OR (Telematic) OR (tele-matic) OR (telemanagement) OR (Tele-management) OR (tele-nursing) OR (telenursing) OR (teleservice) OR (tele-service) OR (tele-care) OR (telecare) OR (Tele-home) OR (telehome) OR (Tele-conferenc*) OR (Teleconferenc*) OR (Teleconsult) OR (Tele-consult) OR (telecommunication) OR (tele-communication) OR (Remote Sensing Technolog*) OR (wearable*) OR (wearable device) OR (wearable sensor)) |

Search in Psycinfo: 0

5) Web of Science

| **S/No.** | **Key themes** | **Terms** |
| --- | --- | --- |
| 1 | Type 2 Diabetes Mellitus | ((Diabetes mellitus) OR (Diabetes mellitus, type 2) OR (T2DM) OR (Type 2 diabetes mellitus) OR (NIDDM) OR (non-insulin dependent diabetes mellitus)) |
| 2 | Caregivers | ((Caregiver) OR (caregiver*) OR (carer) OR (family) OR (care giver) OR (spouse caregiver) OR (family caregiver)) |
| 3 | Remote management | ((Telemedicine) OR (telehealth) OR (Telemonitor) OR (tele-monitor) OR (Telemonitoring) OR (Tele-monitoring) OR (ehealth) OR (electronic health) OR (e-health) OR (mobile health) OR (remote management) OR (remote monitoring) OR (remote consultation) OR (mhealth) or (m-health) OR (digital health) OR (Telematic) OR (tele-matic) OR (telemanagement) OR (Tele-management) OR (tele-nursing) OR (telenursing) OR (teleservice) OR (tele-service) OR (tele-care) OR (telecare) OR (Tele-home) OR (telehome) OR (Tele-conferenc*) OR (Teleconferenc*) OR (Teleconsult) OR (Tele-consult) OR (telecommunication) OR (tele-communication) OR (Remote Sensing Technolog*) OR (wearable*) OR (wearable device) OR (wearable sensor)) |

**Supplementary File 2. Risk of bias assessment for included studies**

1. Randomized controlled trials

| **Study (Year)** | **Domain 1: Risk of bias from the randomization process** | **Domain 2: Risk of bias due to deviations from the intended interventions** | **Domain 3: Missing outcome data** | **Domain 4: Risk of bias in measurement of the outcome** | **Domain 5: Risk of bias in selection of the reported result** | **Overall Risk of bias** |
| --- | --- | --- | --- | --- | --- | --- |
| Piette JD et al (2016) | Some concerns | Some concerns | Low | Some concerns | Low | Moderate |
| Gomes LC et al (2017) | Some concerns | Low | Low | Low | Low | Low |
| Burner E et al (2017) | Some concerns | Some concerns | Low | High | Low | Moderate |
| Piette JD et al (2013) | Some concerns | Some concerns | Low | Some concerns | Low | Moderate |

**b) Observational studies**

| Study (Year) | 1. Was the research question or objective in this paper clearly stated? | 2. Was the study population clearly specified and defined? | 3. Was the participation rate of eligible persons at least 50%? | 4. Were all the subjects selected or recruited from the same or similar populations (including the same time period)? Were inclusion and exclusion criteria for being in the study prespecified and applied uniformly to all participants? | 5. Was a sample size justification, power description, or variance and effect estimates provided? | 6. For the analyses in this paper, were the exposure(s) of interest measured prior to the outcome(s) being measured? | 7. Was the time frame sufficient so that one could reasonably expect to see an association between exposure and outcome if it existed? | 8. For exposures that can vary in amount or level, did the study examine different levels of the exposure as related to the outcome (e.g., categories of exposure, or exposure measured as continuous variable)? | 9. Were the exposure measures (independent variables) clearly defined, valid, reliable, and implemented consistently across all study participants? | 10. Was the exposure(s) assessed more than once over time? | 11. Were the outcome measures (dependent variables) clearly defined, valid, reliable, and implemented consistently across all study participants? | 12. Were the outcome assessors blinded to the exposure status of participants? | 13. Was loss to follow-up after baseline 20% or less? | 14. Were key potential confounding variables measured and adjusted statistically for their impact on the relationship between exposure(s) and outcome(s)? | Overall assessment of bias in each study |
| --- | --- | --- | --- | --- | --- | --- | --- | --- | --- | --- | --- | --- | --- | --- | --- |
| Gambling T et al (2010) | Yes | Yes | Yes | Yes | Yes | Yes | Yes | Yes | Yes | No | Yes | No | Yes | Yes | Low |
| Mayberry LS et al (2019) | Yes | Yes | NA | Yes | Yes | Yes | Yes | Yes | Yes | No | Yes | No | Yes | Yes | Low |
| Zhang Y et al (2021) | Yes | Yes | No | Yes | Yes | Yes | Yes | Yes | Yes | Yes | Yes | Yes | Yes | Yes | Low |
| Aikens JE et al (2015) | Yes | Yes | Yes | Yes | Yes | Yes | Yes | Yes | Yes | No | Yes | No | Yes | Yes | Low |
| Piette JD et al (2013) | Yes | Yes | Yes | Yes | Yes | Yes | Yes | Yes | Yes | No | Yes | No | Yes | Yes | Low |
| Wakefield BJ et al (2017) | Yes | Yes | No | Yes | Yes | Yes | Yes | Yes | Yes | No | Yes | Yes | Yes | Yes | Low |
| Aikens JE et al (2014) | Yes | Yes | Yes | Yes | Yes | Yes | Yes | Yes | Yes | No | Yes | No | Yes | Yes | Low |
