# Supplementary material for: Role of Caregivers in Remote Management of Patients With Type 2 Diabetes Mellitus: Systematic Review of Literature
Source: J Med Internet Res. 2023 Sep 11;25:e46988. doi: 10.2196/46988 (PMC10520771; doi:10.2196/46988)
Supplement: Multimedia Appendix 2 [file jmir_v25i1e46988_app2.docx]

**Multimedia Appendix 2**

**Table S1.** Summary of the 11 studies included into the systematic review.

| **Study author** | **Year** | **Study type** | **Country** | **Cohort size (patients)** | **Type 2 diabetes mellitus age group** | **Study setting** | **Type of caregiver** | **Gender (%, Men)** | **Diabetes mellitus (mean ± SD, years)** | **Baseline HbA1c (mean ± SD, %)** | **Remote monitoring modality** | **Duration of remote monitoring** |  |
| --- | --- | --- | --- | --- | --- | --- | --- | --- | --- | --- | --- | --- | --- |
| **Telephone calls** | | | | | | | | | | | | | |
| Mayberry L.S et. al. (2020) | 2012 | Observational study | United States of America | 313 | Adults | Primary | Family members or friends | 40.2 | 12.6 ± 8.6 | 9.6 ± 1.8 | Phone calls | 6 months |  |
| Gomes LC et. al. (2011) | 2011 | Randomised control trial | Brazil | 190 | Adults | Tertiary | Family members | 43.0 | 15.7 ± 7.82 | Control group: 9.4 (2.0), intervention group: 9.47 (2.01) | Telephone calls | 12 months |  |
| Gambling T et al (2010) | 2002 | Randomised control trial | United Kingdom | 9 | Adults | Primary | Non-medically trained tele-carers | 33.3 | Not stated | 5 had HbA1C >9%, 4 had HbA1c <9% | Telephone calls | 3 years |  |
| Wakefield, BJ et al (2017) | 2009 | Observational study | United States of America | 244 | Adults | Primary | Family members or friends | Control group: 91.9, Intervention group: 97.5, p=0.05 | Not stated | Not stated | Phone calls | 6 months |  |
| **Interactive voice response** | | | | | | | | | | | | | |
| Piette JD et. al. (2016) | 2013 | Randomised control trial | Bolivia | 72 | Adults | Tertiary | Family members or friends | 37.0 | Not stated | Not stated | Interactive voice response | Up to 4 months |  |
| Aikens J.E et. al. (2015) | 2010 | Observational study | United States of America | 301 | Adults | Primary | Family members or friends | 97.0 | Not stated | Not stated | Interactive voice response | 6 months |  |
| James E.Aikens et. al. (2013) | 2010 | Observational study | United States of America | 303 | Adults | Primary | Family members or friends | Control group: 95.8, intervention group: 97.8 | Not stated | Not stated | Interactive voice response | 6 months |  |
| Piette JD et. al. (2013) | 2009 | Randomised control trial | Multi-countries (Honduras, Mexico, United states of America) | 268 | Adults | Primary | Family members or friends | 27.0 | Not stated | Not stated | Interactive voice response | 6 to 12 weeks |  |
| Piette J.D et. al. (2013) | 2009 | Cohort study, retrospective | United States of America | 727 | Adults | Primary | Family members or friends | 70.2 | Not stated | Not stated | Interactive voice response | 12 weeks |  |
| **Text messages** | | | | | | | | | | | | | |
| Burner E et. al. (2018) | 2013 | Randomised control trial | United States of America | 44 | Adults | Tertiary | Family members or friends | 43.0 | Not stated | Control group: 10.1, 95% CI: 9.5, 10.8, Intervention group: 10.4, 95% CI: 9.6, 11.3 | Text messages | 3 months |  |
| **Web application** | | | | | | | | | | | | | |
| Zhang Y et. al. (2021) | 2018 | Cohort study, retrospective | China | 6582 | Adults | Primary | Family member | 56.3 | 2.3 (IQR: 0.1-9.5) | Not stated | Web application | 12 weeks |  |

Legend: IQR – interquartile range
