# Supplementary material for: Role of Caregivers in Remote Management of Patients With Type 2 Diabetes Mellitus: Systematic Review of Literature
Source: J Med Internet Res. 2023 Sep 11;25:e46988. doi: 10.2196/46988 (PMC10520771; doi:10.2196/46988)
Supplement: Multimedia Appendix 3 [file jmir_v25i1e46988_app3.docx]

**Multimedia appendix 3**

**Table S2.** Summary of the outcome measures reported from the 11 studies included into the review.

| **Type of outcomes presented** | **Study author** | **Remote monitoring modality** | **Duration of remote monitoring** | **Type of caregiver** | **Training received by caregiver** | **Role of caregiver** | **Details of the result** |
| --- | --- | --- | --- | --- | --- | --- | --- |
| **Diabetes-related parameters e.g. HbA1c** | Gomes LC et. al. (2011) | Telephone calls | 12 months | Family members | N.A. | To encourage dialogue between the patients and their relatives about the topics related to diabetes. | Clinical laboratory variables such as FPG, HbA1c, Total cholesterol, HDL, LDL, triglycerides, urea and creatinine were not significantly different between IG patients and CG patients at 1 year mark. (p>0.05) |
|  | Burner E et. al. (2018) | Text messages | 3 months | Family members or friends | N.A. | To be a source of social support while caring for the patient. | There was no significant improvement in mean HbA1C between IG v.s. CG (p>0.05) |
|  | Gambling T, Long A (2010) | Telephone calls | 3 years | Non-medically trained tele-carers | Supported by diabetes specialist nurse | To provide advice to the patients on their diabetes care. | There was no significant improvement in mean HbA1C between IG v.s. CG (p>0.05) |
|  | Mayberry L.S et. al. (2020) | Telephone calls | 6 months | Family members or friends | N.A. | To support the patient's diabetes self-management. | IG with frequent contact was associated with significantly better HbA1c among patients with an in-home supporter but with worse HbA1c among patients without an in-home supporter (interaction β=−0.45, p=0.005) |
|  | Zhang Y et. al. (2021) | Web application | 12 weeks | Family member | N.A. | View patients' blood glucose records and diabetes education course learning records, take the diabetes education courses, and participate in 2-way communications with the patients through the family portal. | Patients in IG reported significantly better mean fasting blood glucose and postprandial blood glucose at week 12 (p≤0.05)  A significantly higher proportion of IG patients achieved both fasting blood glucose <7mmol/L and postprandial blood glucose<10mmol/L than CG patients (p=0.02) |
|  | James E.Aikens et. al. (2013) | Interactive voice response | 6 months | Family members or friends | Participating caregivers underwent DVD-based communication training using motivational interviewing principles. | To receive emailed summaries of each completed call along with structured suggestions on supporting the patient's diabetes self-management and to care for the patient from outside of the patient's residence. | IG were less likely to report frequent high blood glucose compared to CG (p = 0.021) |
| **Medication adherence** | Mayberry L.S et. al. (2020) | Phone calls | 6 months | Family members or friends | N.A. | To support the patient's diabetes self-management. | Patients in the IG had significantly better medication adherence compared CG, (AOR = 1.19, p = 0.029) |
|  | Aikens J.E et. al. (2015) | Interactive voice response | 6 months | Family members or friends | N.A. | To receive emailed summaries of each completed call along with structured suggestions on supporting the patient's diabetes self-management and to care for and communicate with the patient remotely. | IG reported improvements in Morisky Medication Adherence Scale pre and post intervention (95% confidence interval: -0.42 to -0.18, p<0.001). |
| **Diabetes-related symptoms and distress** | Piette JD et. al. (2016) | Interactive voice response | Up to 4 months | Family members or friends | N.A. | To talk with their patient-partner once a week to review the information provided on how to assist his or her patient-partner and to address issues identified through the Interactive Voice Response calls. | Patients in the IG were significantly less likely to spend days in bed due to illness than the CG (p=0.029)  Patients in the IG were significantly more likely to report excellent health during their Interactive Voice Response calls than the CG (p=0.034) |
|  | Mayberry L.S et. al. (2020) | Phone calls | 6 months | Family members or friends | N.A. | To support the patient's diabetes self-management. | Greater CarePartner closeness was associated with a significantly greater odds of lower diabetes distress (β=0.14, p=0.012) |
|  | Aikens J.E et. al. (2015) | Interactive voice response | 6 months | Family members or friends | N.A. | To receive emailed summaries of each completed call along with structured suggestions on supporting the patient's diabetes self-management and to care for and communicate with the patient remotely. | IG reported significant improvements in depressive symptoms and diabetes-related distress compared to CG (p<0.001) |
| **Quality of life** | Burner E et. al. (2018) | Text messages | 3 months | Family members or friends | N.A. | To be a source of social support while caring for the patient. | Physical activity score (mean Godin leisure time) did not show significant improvements between groups (p>0.05)    Diabetes-related quality of life (PAID scale) did not show significant improvements between groups (p>0.05) |
|  | Aikens J.E et. al. (2015) | Interactive voice response | 6 months | Family members or friends | N.A. | To receive emailed summaries of each completed call along with structured suggestions on supporting the patient's diabetes self-management and to care for and communicate with the patient remotely. | IG reported significant improvements in physical function compared to CG (p<0.001) |
| **Healthcare utilization** | Wakefield, BJ; Vaughan-Sarrazin, M (2017) | Phone calls | 6 months | Family members or friends | N.A. | To help the Veteran patients with their diabetes care. | IG were more likely to have one or more hospitalizations compared to CG (p=0.001) |
| **Patient satisfaction** | Piette JD et. al. (2013) | Interactive voice response | 6 to 12 weeks | Family members or friends | N.A. | To participate with the patient in listening to feedback on patient's status, changes in health status, and what the caregiver could do to support the patient's self-care. To apply it in the care of the patient. | There was a significant percentage (75.2%) of patients reported improvements in symptom monitoring by "a lot" (p=0.04) |
| **Compliance to remote monitoring** | Piette JD et. al. (2016) | Interactive voice response | Up to 4 months | Family members or friends | N.A. | To talk with their patient-partner once a week to review the information provided on how to assist his or her patient-partner and to address issues identified through the Interactive Voice Response calls. | The call completion rate was significantly higher in the IG patients compared to CG patients (62.0% versus 44.9%; p < 0.047). |
|  | Burner E et. al. (2018) | Text messages | 3 months | Family members or friends | N.A. | To be a source of social support while caring for the patient. | There was a significant increase in the self-monitoring frequency for glucose (IG: +1.6 days/week; CG: -2 days/week, p=0.02) |
|  | Zhang Y et. al. (2021) | Web application | 12 weeks | Family member | N.A. | View patients' blood glucose records and diabetes education course learning records, take the diabetes education courses, and participate in 2-way communications with the patients through the family portal. | There was a significantly greater frequency of self-monitored of blood glucose at week 12 in the IG than CG (p=0.05)  There was a significantly greater number of diabetes education courses completed in 12 weeks in the IG than CG (p<0.001) |
|  | Piette J.D et. al. (2013) | Interactive voice response | 12 weeks | Family members or friends | N.A. | Receive automated emails with feedback about patient's status and how they could support the patient's self-management. Then, using the feedback to support their patient. | IG were significantly more likely to complete interactive voice response assessments compared to control  (adjusted odds ratio, 1.37; 95% confidence interval, 1.07-1.77, p=0.001) |
|  | James E.Aikens et. al. (2013) | Interactive voice response | 6 months | Family members or friends | Participating caregivers underwent DVD-based communication training using motivational interviewing principles. | To receive emailed summaries of each completed call along with structured suggestions on supporting the patient's diabetes self-management and to care for the patient from outside of the patient's residence. | IG were more likely to regularly check their blood pressure (p=0.017) |

Legend: CG – control group; IG – intervention group
